# 

**Published:** 1898-05

**Authors:** 


### CONTENTS.

ORIGINAL COMMUNICATIONS. PAGE

Method of using Soft Foil. By BENJAMIN LORD, D.D.S., New York . . . . . 265
The Preparation of the Mouth for an Artificial Denture preparatory to taking an Impression. By ROBERT H. NONES, D.D.S., Philadelphia . . . . . . . . . . 270
An Artificial Piece for an Ear. By WILLIAM DUNN, D.D.S., Florence, Italy . . 277
Etruscan Bridge-Work. By HENRY H. BURCHARD, M.D., D.D.S. . . . . . . . 278

ABSTRACTS AND TRANSLATIONS.

Instrument Nomenclature with Reference to Instrumentation. By G. V. BLACK, M.D., D.D.S., Sc.D., Chicago . . . . . . . . . . . . . . . . . . . . . . 279

REPORTS OF SOCIETY MEETINGS.

The New York Institute of Stomatology . . . . . . . . . . . . . . . . . . 299

EDITORIAL.

The Need for Original Investigation . . . . . . . . . . . . . . . . . . . . 314
The National Dental Association, Division of the East . . . . . . . . . . . . . 317
The Recently Enacted Law of New Jersey . . . . . . . . . . . . . . . . . . 317
Standard of Entrance raised . . . . . . . . . . . . . . . . . . . . . . . . 320

DOMESTIC CORRESPONDENCE.

What is Dentistry? . . . . . . . . . . . . . . . . . . . . . . . . . . . . 320
Election of Delegates to National Association . . . . . . . . . . . . . . . . 325
Burning by Röntgen Rays . . . . . . . . . . . . . . . . . . . . . . . . . 326

OBITUARY.

Dr. Albert Nelson Chapman . . . . . . . . . . . . . . . . . . . . . . . . 327

NOTES AND COMMENTS . . . . . . . . . . . . . . . . . . . . . . . . . . 328

CURRENT NEWS.

Massachusetts Dental Society . . . . . . . . . . . . . . . . . . . . . . . 330
National Dental Association, Division of the East . . . . . . . . . . . . . . . 331
Harvard Odontological Society . . . . . . . . . . . . . . . . . . . . . . . 332

